# A putative design for the electromagnetic activation of split proteins for molecular and cellular manipulation

**DOI:** 10.3389/fbioe.2024.1355915

**Published:** 2024-03-28

**Authors:** Connor J. Grady, E. Alejandro Castellanos Franco, Jory Schossau, Ryan C. Ashbaugh, Galit Pelled, Assaf A. Gilad

**Affiliations:** ^1^ Department of Biomedical Engineering, Michigan State University, East Lansing, MI, United States; ^2^ Department of Chemical Engineering and Materials Science, Michigan State University, East Lansing, MI, United States; ^3^ Department of Computer Science and Engineering, Michigan State University, East Lansing, MI, United States; ^4^ Department of Electrical and Computer Engineering, Michigan State University, East Lansing, MI, United States; ^5^ Department of Mechanical Engineering, Michigan State University, East Lansing, MI, United States; ^6^ Department of Radiology, Michigan State University, East Lansing, MI, United States

**Keywords:** split proteins, split enzymes, magnetoreception, bioluminescence, thymidine kinase

## Abstract

The ability to manipulate cellular function using an external stimulus is a powerful strategy for studying complex biological phenomena. One approach to modulate the function of the cellular environment is split proteins. In this method, a biologically active protein or an enzyme is fragmented so that it reassembles only upon a specific stimulus. Although many tools are available to induce these systems, nature has provided other mechanisms to expand the split protein toolbox. Here, we show a novel method for reconstituting split proteins using magnetic stimulation. We found that the electromagnetic perceptive gene (EPG) changes conformation due to magnetic field stimulation. By fusing split fragments of a certain protein to both termini of the EPG, the fragments can be reassembled into a functional protein under magnetic stimulation due to conformational change. We show this effect with three separate split proteins: NanoLuc, APEX2, and herpes simplex virus type-1 thymidine kinase. Our results show, for the first time, that reconstitution of split proteins can be achieved only with magnetic fields. We anticipate that this study will be a starting point for future magnetically inducible split protein designs for cellular perturbation and manipulation. With this technology, we can help expand the toolbox of the split protein platform and allow better elucidation of complex biological systems.

## Introduction

An ongoing effort in chemical biology is directed toward creating tools that can manipulate cellular systems with molecular precision. A common technique researchers use to address the challenge of temporal and spatial activation of proteins is the split protein approach. This method generally uses a functional protein that has been fragmented in a way that allows the split parts to reconstitute and regain native function. Split proteins have been widely used to study protein–protein interactions where researchers fuse split reporter proteins to two interacting proteins and observe their fluorescence (Hu and Kerppola, 2003; Romei and Boxer, 2019) or bioluminescence (Paulmurugan and Gambhir, 2003; Dixon et al., 2016). More recently, the split protein method has been expanded to control protein function under specific stimulus such as light (optogenetics) (Kawano et al., 2016; Yu et al., 2020) or chemicals (chemogenetics) (Han et al., 2019). These approaches have been used to regulate cellular functions such as transcription (Zetsche et al., 2015; Nihongaki et al., 2019) and enzymatic activity (Shekhawat and Ghosh, 2011; Cubillos-Ruiz et al., 2022), as well as being used in the creation of cellular circuits (Gao et al., 2018; Fink et al., 2019), demonstrating their usefulness for studying complex biological systems.

The current methodologies for controlling split proteins have been well established; nevertheless, expanding the split protein toolbox to include tools that respond to different stimuli could lead to further discoveries. One such stimulus with the potential for broad impact is magnetism. Magnetic fields represent a non-invasive stimulus with equal distribution that has no limitations of penetration depth. Their “on”/“off” functionality associated with electromagnetic coils also allows for precise control. Several approaches have shown effective magnetic activation of cellular functions by ion channel interactions (Huang et al., 2010; Stanley et al., 2012; Stanley et al., 2015; Wheeler et al., 2016). A novel electromagnetic perceptive gene (EPG) from the glass catfish *Kryptopterus vitreolus* has also been shown to have a response to changes in magnetic fields (Krishnan et al., 2018; Cywiak et al., 2020; Hwang et al., 2020; Metto et al., 2022).

In this study, we combine the split protein approach with the EPG protein to create the first magnetogenetic activatable biological hinge. This system is different from the standard split protein systems, where two interacting proteins are needed for the reconstitution of the fragments. Both fragments of the EPG split protein system are fused to either end of the EPG protein, and conformational changes in the EPG protein bring the fragments together in the presence of a magnetic field. We tested this with the three established split protein systems of NanoLuc (Zhao et al., 2016), APEX2 (Han et al., 2019), and the herpes simplex virus type-1 thymidine kinase (HSV1-TK) (Massoud et al., 2010). Through these split proteins, we showed the potential of this first-of-its-kind technology and set the foundation for future magnetically activated split protein systems.

## Results

### EPG conformation changes in response to magnetic stimulation

**FIGURE 1:**
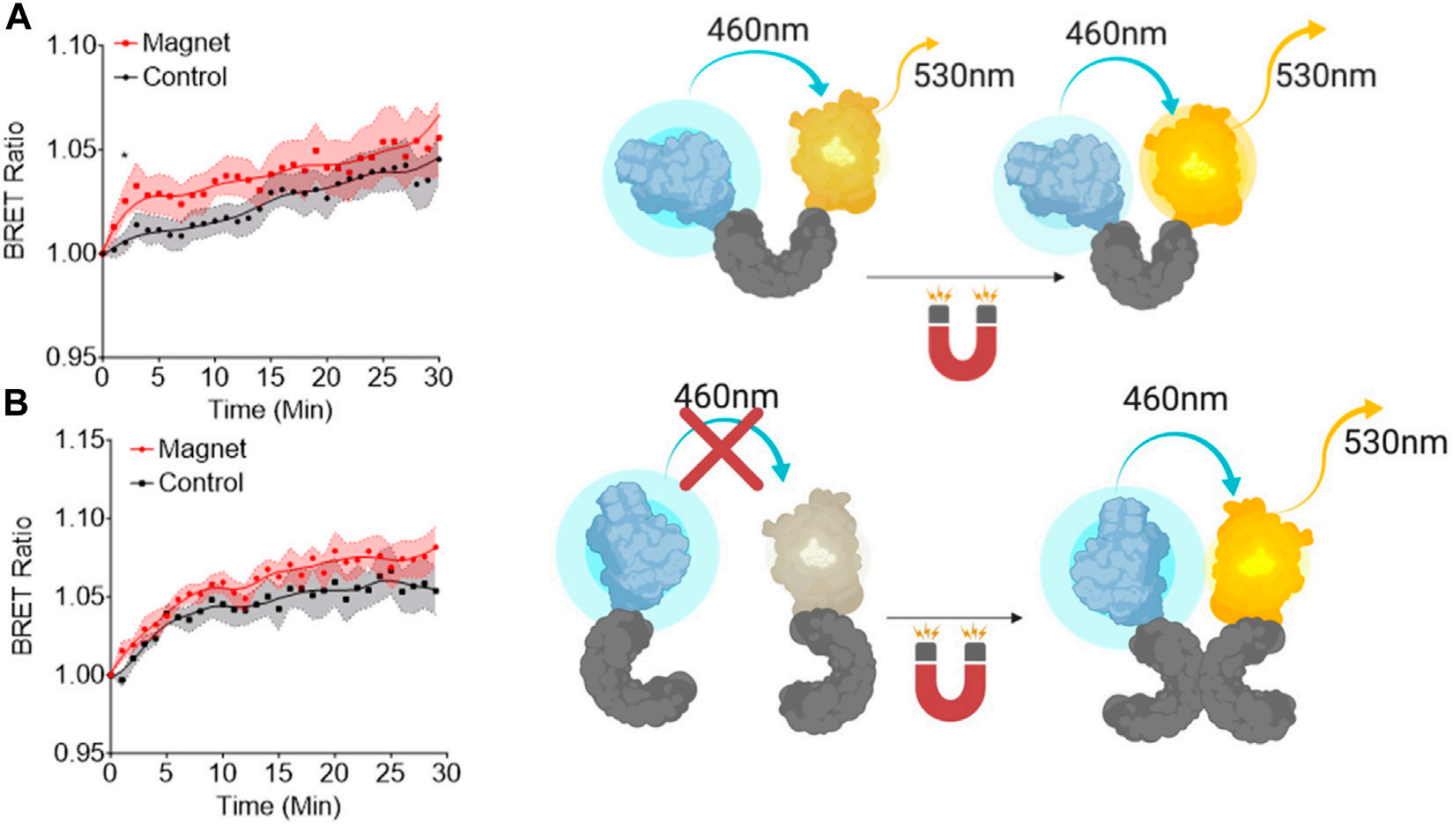
Bioluminescent resonance energy transfer studies of EPG conformational changes. **(A)** A single copy of EPG (gray) cloned between NanoLuc (blue) and mVenus (yellow). **(B)** A copy of the EPG was fused to NanoLuc followed by an internal ribosome entry site (IRES) and an EPG fused to an mVenus to express both constructs on the same plasmid. Readings were taken at 530 nm and 460 nm every minute for 30 min with or without constant static magnetic stimulation. Readings were normalized to the last read before stimulation. Fit line in each graph is a lowess smoothing to show the relationship between the groups. Data are shown as mean ± S.E.M. N = 15 wells were analyzed for the single and N = 9 for the EPG IRES experiments. Statistical analysis was performed using an unpaired *t*-test with Welch’s correction at the saturation time point of each experiment (T = 2, A; T = 7, B). A (*) denotes a *p*-value <0.05.

Bioluminescence resonance energy transfer (BRET) studies are used for determining if conformational changes occur within a protein (Audet et al., 2008; Inagaki et al., 2017). Using this design, we studied if EPG has a conformational change due to static magnetic field (10 mTesla). Previous studies have shown that there was no change in conformation with 25 mTesla when EPG was in a purified form (Krishnan et al., 2018), but this could also be due to not having potential cofactors to help facilitate this change. Because of this, we decided to fuse EPG to the blue-emitting bioluminescent protein NanoLuc and the yellow-emitting fluorescent protein mVenus on the N and C terminals, respectively, and was expressed in HeLa cells. Figure 1A shows that there is a 2.5% signal increase in the group stimulated by magnetic field over the non-stimulated group. The response seen is comparable to other BRET studies of single-protein conformational changes (Audet et al., 2008; Pétigny et al., 2022). We then designed a BRET construct to test if the protein underwent a dimerization event due to magnetic stimulus. This has the EPG fused to NanoLuc on the C terminal followed by an IRES site followed by EPG fused to mVenus (EPG-NanoLuc IRES EPG-mVenus). The group stimulated with the static magnet had a 1.5% increase compared to the control group (Figure 1B). The response from the EPG IRES experiment is not consistent with the standard BRET studies for the protein–protein interaction (Machleidt et al., 2015). The low response implies that the dimerization of EPG is not the mechanism by which EPG works. Collectively, these findings suggest that magnetic stimulation led to a conformational change in the EPG protein.

### EPG BRET constructs are localized in the cytoplasm

**FIGURE 2:**
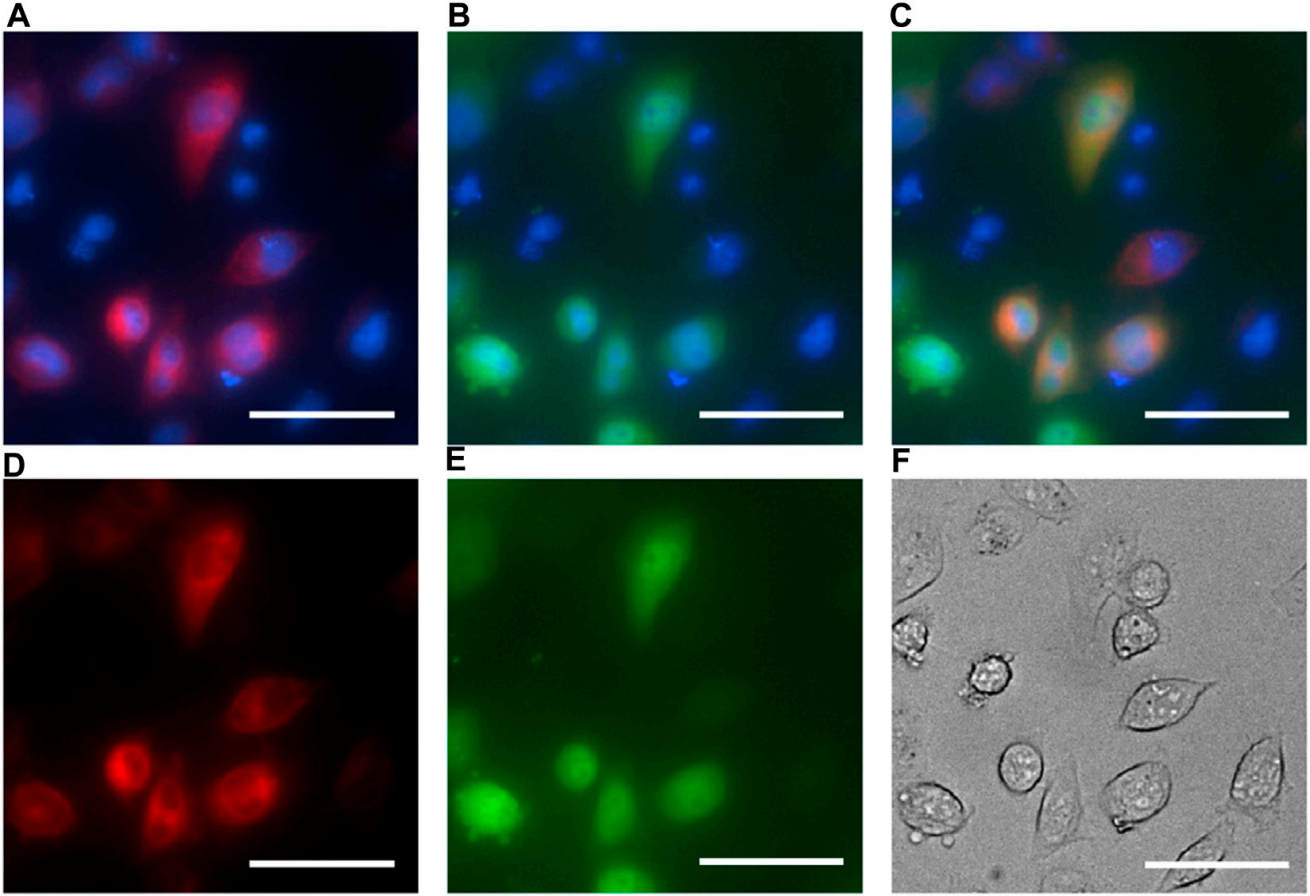
EPG BRET fluorescent imaging for cell localization. HeLa cells co-transfected with the EPG BRET construct and EPG N terminus HaloTag construct and imaged with ×40 magnification. Hoechst dye was used as a nuclear marker and imaged using the DAPI filter (blue: A, B, and C). The EPG HaloTag construct was imaged using a JFX 650 dye with the Cy5 filter overlaid with a nuclear marker **(A)** and without a nuclear marker **(D)**. EPG BRET construct was imaged using the GFP filter overlaid with a nuclear marker **(B)** and without a nuclear marker **(E)**. Merged image of the three channels **(C)** shows an expression of the EPG BRET construct in the cytoplasm, and the EPG HaloTag construct on the cell membrane. **(F)** Phase contrast image of cells. Scale bar = 50 µm.

The BRET constructs were cloned in a way that should block the signal sequence and the membrane anchor sequence of the EPG. Therefore, we anticipated cytoplasmic expression. To test this hypothesis, we co-expressed the EPG BRET construct and an EPG HaloTag construct that was previously shown to be membrane-anchored in mammalian cells (Ricker et al., 2023). Fluorescent images show the BRET construct was likely expressed in the cytoplasm as opposed to the EPG HaloTag fusion protein mostly observed on the cellular membrane. Figure 2 demonstrates that the EPG BRET construct is a cytoplasmic protein, providing evidence supporting that the membrane and signal sequences were blocked. Moreover, the expression of the EPG split NanoLuc (Supplementary Figure S1) clearly shows that the protein expression is cytoplasmic. These findings indicate that the conformational change that occurs in the cytoplasm also indicates that the magnetoreception of EPG is not dependent on its cellular localization.

### EPG split NanoLuc expressed in *E. coli* exhibits an increase in bioluminescence in response to magnetic stimulation

**FIGURE 3:**
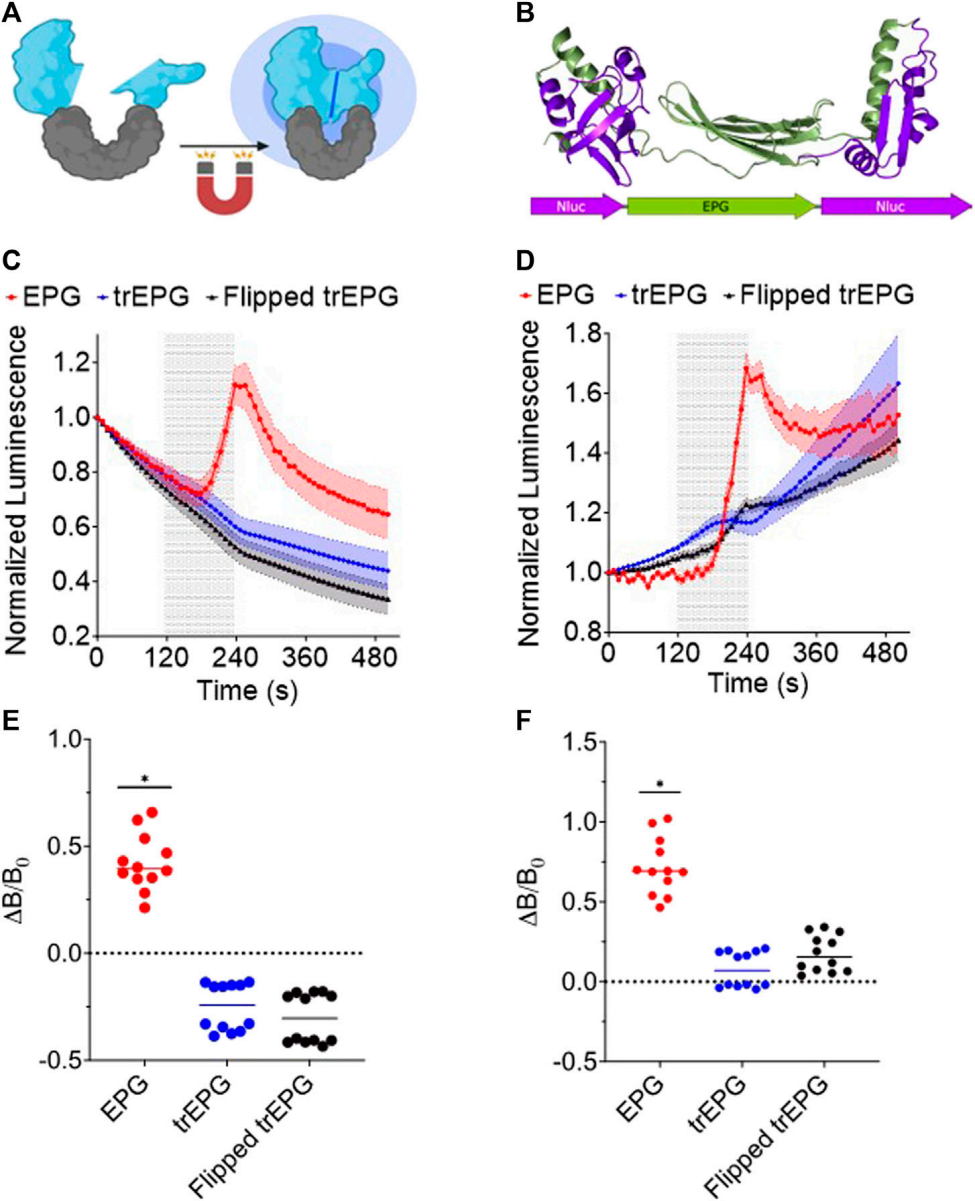
EPG split NanoLuc experiments in *E. coli* BL21 cells. Readings were taken on the IVIS every 10 s with an open filter. Electromagnetic stimulus was applied to the cells for 2 min and shown as a shaded region. **(A)** Illustration of the EPG split NanoLuc construct. **(B)** A model of the EPG split NanoLuc construct. *E. coli* lysate **(C)** and whole-cell *E. coli* **(D)** containing the EPG split NanoLuc (red) showed an increase in luminescence in contrast to EPG-truncated (Blue) and --flipped EPG (Black). Data are shown as mean ± S.E.M. Change in luminescence from before and after stimulus of each well in lysate **(E)** and whole-cell **(F)** groups is shown with a line at median. The results shown are duplicate experiments with N = 6 wells in each trial. Statistical significance was calculated using an unpaired *t*-test with Welch’s correction. A (*) denotes *p*-value <0.05.

Building upon the split protein concept and on the magnetoresponsive properties of the EPG, we developed a new platform that allows remote activation of a protein or enzyme using electromagnetic fields (EMFs). The principle for this tool is cloning the EPG between two parts of a split protein or between two enzymes/proteins that need close proximity for activation (Figure 3A). Here, we split NanoLuc (171 amino acids) into two fragments at amino acid sites 65 and 66. The 1–65 and 66–171 fragments were fused to the EPG N and C terminals, respectively. We chose this split site based on previous reports (Zhao et al., 2016) (Figure 3B). A truncated version of this construct was created by removing the signal sequence and membrane anchor sequence of the EPG. Another construct was created using the reverse nucleotide sequence of the truncated EPG, and this was referred to as flipped trEPG. When exposed to EMF, when measured in the cell extract, the EPG construct displayed a 39.4% ± 41.4% compared to control truncated or reverse-truncated EPG (Figure 3C). Under the same condition but when measured in the intact cells, it showed up to 68.7% ± 24.6% increase in luminescence in contrast to control constructs (Figure 3D). We quantified the change in luminescence due to magnetic stimulation by subtracting the luminescence at the last read of stimulation by the last read before stimulation and then dividing by the last read before stimulation. The results of the changes in luminescence from each well from the lystate (Figure 3E) and whole-cell (Figure 3F) groups show significant increases in luminescence from the EPG group when compared to the trEPG and Flipped trEPG groups. These results are the first demonstration that a split protein can be brought together by the conformational change of the EPG. Thus, the EPG can act as a magnetically activatable hinge.

### Remote magnetic control of peroxidase activity using EPG split APEX2

**FIGURE 4:**
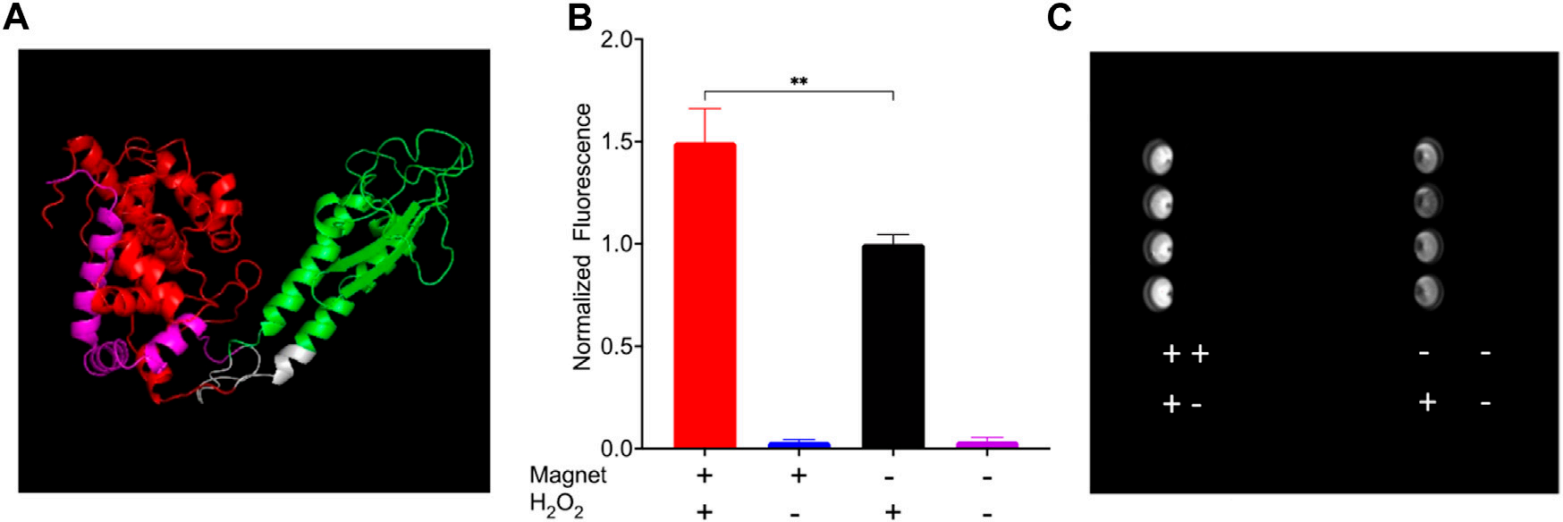
HEK 293FT cells expressing EPG split APEX2 show an increase in fluorescence in response to magnetic field. All wells were treated with Amplex UltraRed reagent and the four combinations of with or without magnetic stimulus and H_2_O_2_ for 30 min. **(A)** Predicted structure of EPG split APEX2 with EPG (green), AP fragment (Red), EX fragment (magenta), and linkers (white). **(B)** Endpoint results of cells treated with all combinations of static magnetic stimulus and hydrogen peroxide (n = 4 independent experiments). **(C)** Image of a plate taken with a Cy3 filter after experiment for the detection of resorufin accumulation. Statistical analysis was performed using an unpaired *t*-test with Welch’s correction. The (**) denotes *p*-value <0.01.

To demonstrate that the EPG split approach can be used as a platform technology, we used a split APEX2 peroxidase (Han et al., 2019). This system allows simplified demonstration of the concept that EMF can control an enzymatic reaction, and the output can be measured directly with colorimetric or fluorescent reaction with any standard plate reader or potentially even a microscope. HEK 293FT cells expressing EPG split APEX2 treated with both static magnetic stimulus and hydrogen peroxide displayed a clear increase in fluorescence (150% ± 16%; Figure 4) compared to the cells that did not experience magnetic stimulation. These results show a statistically significant increase in peroxidase activity in response to 30 min of exposure to static magnetic field (−150 mT). We also repeated this experiment at room temperature and 37°C and found similar results (Supplementary Figure S2). These findings indicate that the EPG protein can be used as magneto-switch to activate multiple enzymes.

### EPG split HSV1-TK ganciclovir-mediated cell death

**FIGURE 5:**
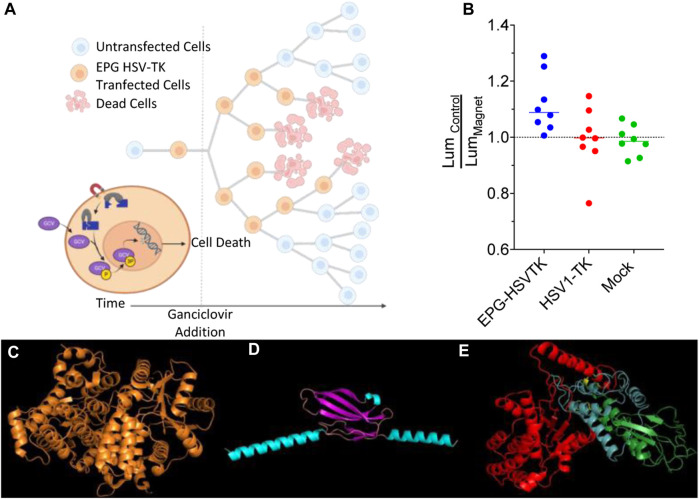
Ganciclovir-mediated cell death; control vs. magnet. **(A)** Schematic representation of the experimental process and design. **(B)** Ratio of average control cell luminescence to magnetic-stimulated cell luminescence over the course of eight experimental replicates. **(C)** Structure of HSV1-TK, **(D)** predicted structure of EPG with core structure (purple) and the signal sequence and membrane anchor sequence (teal), and **(E)** predicted structure of EPG split HSV1-TK with N-terminal HSV1-TK (red), EPG (green), and C-terminal HSV1-TK (blue).

To demonstrate potential therapeutic and diagnostic usage of an EPG split protein, we decided to create an EPG split herpes simplex virus thymidine kinase. This construct was based on the previously split sr39 mutant of HSV1-TK (Massoud et al., 2010). To test the EPG split HSV-TK, we used the proven suicide gene therapy combination of HSV1-TK and ganciclovir (GCV), where the HSV-TK phosphorylates the GCV, allowing other cellular enzymes to further phosphorylate and incorporate the GCV triphosphate into the DNA, causing cell death (Alon et al., 2018). To perform the cell uptake assay of the GCV, 4T1-Luc2 cells transfected with either EPG split HSV1-TK, wildtype HSV1-TK, or a mock transfection were subjected to 0.15 mg/mL of GCV for 72 h. The 4T1-Luc2 cell line expresses the gene Luc2, an ATP-dependent luciferase. Therefore, cell viability was quantified by directly measuring luminescence after the 72 h of incubation with GCV (Figure 5A). A linker screening was performed with three combinations of an EPG without the signal sequence and membrane localization signal and four full-length EPG constructs. These constructs were cloned with either flexible (GGGGS) or rigid (PAPAP) linkers between the EPG and split fragments of the HSV1-TK and then were tested for magnetic response after treatment with GCV. After the initial screening, two constructs showed potential, and the full-length EPG with a flexible first linker and a rigid second linker was chosen due to a lower basal activity (Supplementary Figure S3).

To test the construct with full-length EPG with a flexible first linker and rigid second linker further, eight experimental replicates were performed comparing magnetic stimulated cells and non-stimulated cells. Due to the differences in final cell numbers in each replicate, we decided to use two statistical tests: a condition significance, how likely it is to observe all eight replicates of average cell growth showing inhibition if the experimental magnetic condition had no effect, and replicate significance, how likely it is to observe, for each replicate, the particular difference of means if the experimental magnetic condition had no effect and the data for both conditions had come from the same distribution (Supplementary Material). In each of these replicates, the mean of luminescence of the EPG-HSV1-TK magnetic-stimulated cells was lower than that of the control EPG-HSV1-TK cells with the average percent change between these groups of 10% (Figure 5B; Supplementary Figures S4A–H), and the probability for such event is 0.00039 (Supplementary Figure SI5). This was not the case with the HSV1-TK () and mock-transfected (Supplementary Figures S4Q–X) groups, which have an average of 3.6% and 1.3%, respectively. In both these control groups, there was no consistent trend of cell viability due to magnetic stimulation as both groups showed three experiments with lower average cell viability and five experiments of increased cell viability in the presence of magnetic field (Figure 5B; the probability for such event is 0.375; Supplementaary Figures S5, S6, S7). Therefore, it appears that even in a complex system such as EPG split HSV1-TK and GCV, a significant yet small effect of magnetic field can be measured. Together with the other experiments, our finding implies that the EPG can be used as a bio-magnetic switch for remote magnetic activation of enzymes.

## Discussion

We demonstrated a novel method to control split proteins using magnetic fields. To the best of our knowledge, we are the first to create this kind of technology. We were able to see an effect of the magnetic field in each of the constructs shown, but challenges arose in some of the constructs. For example, in the EPG HSV1-TK experiments, the high number of cell divisions in each of the experimental replicates led to a small measurable effect. Because of the design of this experiment, many factors attributed to this high number of cell divisions (Alon et al., 2018) (Figure 5A). Since this is a suicide gene therapy, the number of starting cells highly influenced the outcome of this experiment. This, coupled with transient transfection and the fact that ganciclovir is incorporated during cell division, causes non-transfected cells to take over the cell population. Despite this, we still see a significant effect of the magnetic stimulated groups compared to the control groups.

Nevertheless, remote-controlled HSV1-TK has tremendous therapeutic and diagnostic importance for positron emission tomography (PET) (Serganova et al., 2007; Keu et al., 2017) and magnetic resonance imaging (MRI) (Bar-Shir et al., 2013a; Bar-Shir et al., 2013b). Further optimization of the EPG, the linker using HSV1-TK mutants (Allouche-Arnon et al., 2022), or alternative nucleoside kinases (Likar et al., 2010; Bar-Shir et al., 2018) might improve the construct’s performance. This will allow imaging in *in vivo* systems.

Several options exist for the clinical application of EPG split HSV1-TK; one example is engineering cytotoxic T lymphocytes (CTLs). Either using the HSV1-TK as a therapeutic gene or as a reporter gene in combination with other therapeutic genes such as interleukin-13 (IL-13) was demonstrated in a clinical trial (Keu et al., 2017). In another clinical trial, HSV1-TK was used as a reporter for adenoviral-mediated transgene expression in liver cancer patients (Penuelas et al., 2005). In all cases of using HSV1-TK, there is a concern about the “off-target” effect where the target genes, whether mediated by viruses or cells, can end in a location other than the target. Therefore, having means for remote control of the HSV1-TK activity is crucial. In this case, a magnetic field can control the enzyme activity in a special and temporal manner. Indeed, the conditions—such as magnetic field duration—for optimal control of EPG split HSV1-TK must be determined for each scenario. Nevertheless, this approach is likely to reduce side effects.

Interestingly, we found that in the split NanoLuc experiments, keeping the signal sequence and membrane anchor sequence is critical. Although those sequences do not play a role in the magnetoreception as they are believed to be removed in the post-translational processing of EPG, they act as linker sequences for the split proteins. When removed, the split protein did not respond to magnetic fields and provided a much higher luminescence. Thus, we theorize that the split halves have reassembled independently of the function of the EPG. Similar phenomena were previously reported for some split proteins (Dolberg et al., 2021). Because of the hinge design of these constructs, the close proximity of split halves needs to be considered during the design process.

The mechanism by which the EPG responds to magnetic fields is still currently under investigation (Ricker et al., 2022). Although this study provides some insights into the potential mechanisms, that was not the focus of this study. We believe that further progression of structure, function, and applications will be complementary to each other in both discovery of the full mechanism and development of new and improved magnetogenetic tools.

This work creates a new platform to control split proteins. This study was focused on establishing the platform rather than optimizing each individual construct created. No linker optimization was carried out for the EPG BRET, EPG split NanoLuc, and EPG split APEX. In contrast, for the EPG split HSV1-TK, seven linkers were tested. The linkers have an immense importance in aligning the two halves of the split enzyme. Regardless, we were still able to see the magnetic effect in each of these constructs. Therefore, this work sets the future of using magnetic fields as an induction mechanism to control split proteins.

In essence, the technology has the potential to control enzymes remotely and non-invasively. This can be particularly useful in cases where precise timing is crucial, such as the release of specific neurotransmitters (e.g., dopamine or serotonin) or hormones (e.g., insulin). Additionally, this technology could aid in “closed-loop stimulation,” where physiological changes can directly activate magnetoreceptive enzymes. An example of this would be epilepsy, where the seizure itself triggers an “electrical storm” that activates the EPG, ultimately reducing the severity of the seizure (Metto et al., 2023). One drawback of this technology is that it requires optimization of each enzyme, usually by testing different split sites or linkers. Although this process can be tedious, advancements in computational protein structure prediction should make it more efficient in the future.

## Materials and methods

### Cell lines

The cell lines present in this study (HEK 293FT and 4T1 Luc2) were obtained from the American Type Culture Collection (ATCC).

### Plasmid construction

A list of constructs used in this study is provided in Supplementary Material. For cloning, PCR fragments were amplified using Platinum SuperFi II Polymerase (Invitrogen). The fragments were assembled by either NEBuilder HiFi Assembly (New England Biolabs, NEB) or TOPO directional cloning (Invitrogen). Assembled products were heat-shock transformed to 5-alpha (NEB), TOP10 (Invitrogen), or BL21 Star™ (DE3) (Invitrogen) bacteria.

### Bioluminescent resonance energy transfer assay

HeLa cells were split to 70% confluency in a 6-well plate. The following day, the cells were transfected with plasmid DNA, according to the Lipofectamine 3000 protocol. The transfection efficiency was checked under the Keyence microscope using the GFP filter. The cells were then split to black-walled clear-bottom plastic 96-well plates. A stock solution (50 mM) of h-Coelenterazine (h-CTZ, Nanolight Technology) was prepared by adding 25 uL of solution to dried h-CTZ powder. A working concentration of 5 uM was made by diluting the h-CTZ stock solution in FluoroBrite DMEM (Gibco).

Prior to measurements, culture media were aspirated from the cells and replaced with h-CTZ containing media. The plate was then put into a VICTOR Nivo (Perkin Elmer) plate reader. Reads were taken every minute for 15 min from the bottom of the plate using 480/30 nm and 540/30 nm filters. The plate was then taken out, and static magnets were put into wells for magnet samples; then, the plate was placed back in the reader, and readings were taken every minute for 15 min. A ratio of 540/480 was used to calculate BRET efficiency.

### NanoLuciferase assay

Plasmids containing NanoLuciferase constructs were transformed into BL21 *E. coli* cells. Colonies were picked and grown in MagicMedia (Invitrogen) expression media overnight at 37°C. After overnight expression, the cells were pelleted by centrifugation, followed by resuspension in PBST and were sonicated using 10 s on 20 s on pulses for 2–3 min to create cell lysates.

For IVIS (Perkin Elmer) imaging, 25 uL of cells or cell lysate were added to the 96-well plate, followed by 150 uL of LB broth with 5 uM h-CTZ. Then, 15 min after the addition of h-CTZ, IVIS images were captured using a 1 s exposure time with an open emission filter and an F stop of 1, which allowed us to capture an image every 10 s. After 2 min of imaging, an electromagnetic coil (35 mTesla field strength) surrounding the 96-well plate (Ashbaugh et al., 2021) was turned on, and samples were placed under electromagnetic stimulation for a 2 min period at which the magnet was turned off and images were captured for another 6 min. Images were analyzed using the Living Image software (Perkin Elmer).

### Amplex ultrared assay

HEK 293FT cells were grown to 70%–90% confluency and transfected in a 6-well plate, according to the manufacturer’s protocol (Lipofectamine 3000). After 24 h of transfection, the cells were split into black-walled 96-well plates and left to grow for 18–24 h. The cells were then moved to ice, and media were replaced with a solution of 50 uM Amplex UltraRed (Life Technologies) with 0.02% (6.7 mM) H202 in PBS. Cells with magnet stimulation had static magnets (150–200 mTesla) on the top and bottom of the well plate over the stimulated wells. Stimulation occurred for 30 min and then read on a Cytation 5 plate reader (BioTek) using 530 excitation and 590 emission read settings. Images (Figure 4B) were taken on ChemiDoc (Bio-Rad) using the Cy3 blot function.

### Ganciclovir-mediated cell death

4T1 Luc2 (ATCC) cells were plated at 10,000–20,000 cells per well into 96-well plates. After 8 h, the cells were transfected, according to the manufacturer’s protocol (Lipofectamine 3000). The following day, media were exchanged with media containing 0.15 mg/mL ganciclovir (InvivoGen). Magnet-stimulated cells were then placed under constant magnetic stimulation (−150 mT) for 72 h. After 72 h, viability was measured by exchanging media with FluoroBrite (Invitrogen) supplemented with 0.15 mg/mL d-Luciferin (Gold Biotechnology). Luminescent reads were then taken on a Spark (Tecan) plate reader.

### Software

Creation of the protein models was performed based on structure predictions using the Robetta server (Baek et al., 2021) and RoseTTAFold modeling method. Illustrations used in the figures were created with BioRender.com. Graphs and statistical analysis were performed using GraphPad Prism version 9.4.1 for macOS, GraphPad Software, San Diego, California, United States (www.graphpad.com).

## Data Availability

The original contributions presented in the study are included in the article/Supplementary Material; further inquiries can be directed to the corresponding author.
