# Supplementary material for: A putative design for the electromagnetic activation of split proteins for molecular and cellular manipulation
Source: Front Bioeng Biotechnol. 2024 Mar 28;12:1355915. doi: 10.3389/fbioe.2024.1355915 (PMC11007078; doi:10.3389/fbioe.2024.1355915)
Supplement: Supplementary file 1 [file DataSheet1.pdf]

Supplemental Material

A putative design for electromagnetic activation of split proteins for molecular and cellular manipulation

Supplemental Figures

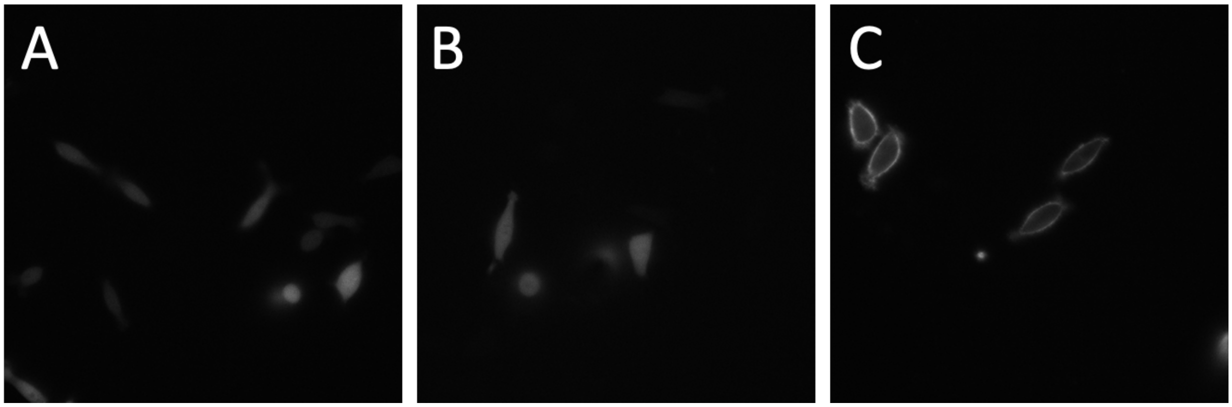

Fig. S1. Cytoplasmic expression of EPG split NanoLuc. Two isoforms of EPG split NanoLuc expressed in HeLa cells A) EPG split NanoLuc with a rigid linker at the N terminus and a flexible linker at the C terminus, and B) EPG split NanoLuc with a flexible linker at the N and rigid linker at the C terminus, respectively. HeLa cells transiently transfected using Lipofectamine 3000 to express the appropriate constructs were cultured on 35 mm glass-bottom dishes in DMEM containing 10% FBS under 5% CO_2 at 37°C. Prior to imaging, the culture medium was replaced by DMEM/F-12 without phenol red. For bioluminescence imaging, cells were incubated with 25µM Fluorofurimazine (Promega) for EPG split NanoLuc (A, B) or 50µM h-Coelenterazine (NanoLight Technology) for membrane-bound glutamate indicator (C) and imaged using an IX71 Inverted Microscope (Olympus). The microscope system was equipped with a UApo/340 40X Oil Iris objective lens (NA = 1.35) and an electron multiplying CCD (EM-CCD) iXON Ultra 888 camera (Oxford Technologies). Exposure time was set to 8 sec for all constructs. In both cases, the expression pattern of EPG split NanoLuc appeared cytoplasmic. This is in contrast to C) cells expressing a membrane-bound glutamate indicator (Petersen et al.[1]). whose expression pattern is limited to the cell membrane.

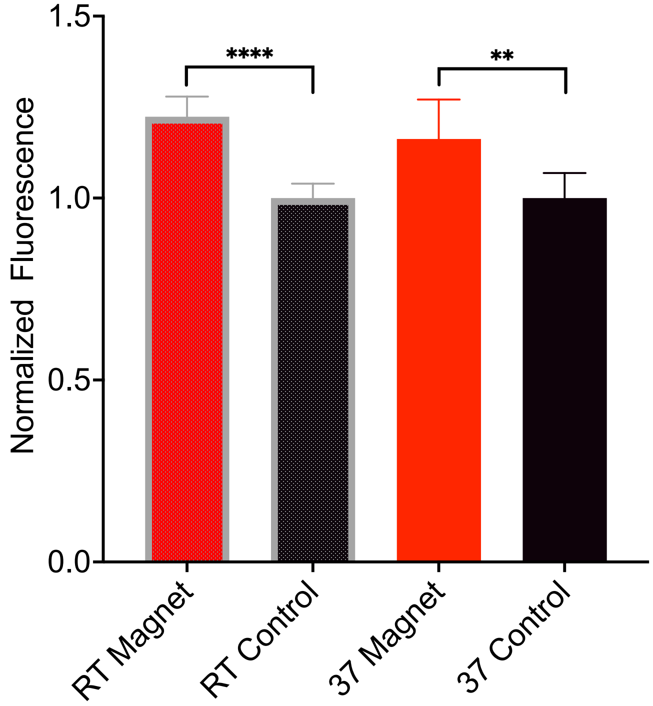

Supplemental Figure 2: EPG split APEX2 temperature variation.
Comparison of the EPG split APEX2 system at room temperature and 37C. Cells were either subjected to magnetic field (red) or no stimulus (black). N=8 biological replicates per group. The (**) denotes p-value <0.01 and the (****) denotes a p-value <0.0001.

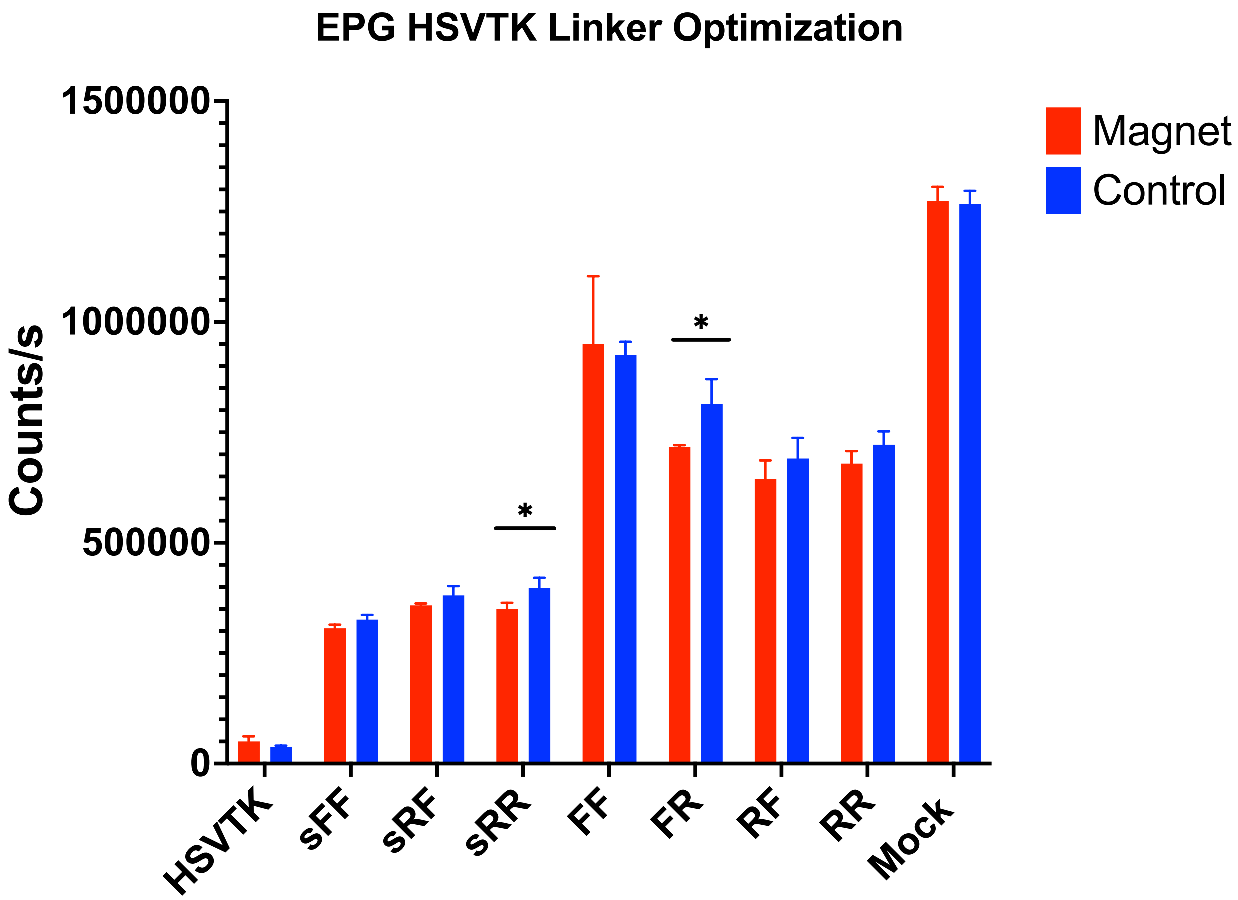

Supplemental Figure 3: Linker Screening of EPG-HSVTK constructs in 4T1 Cells.
Bioluminescent readouts from constructs after 72 hours of GCV treatment with and without magnetic stimulation. The lowercase "s" denotes EPG without signal sequence and membrane sequences. The capital "F" denotes a flexible linker and capital "R" denotes a rigid linker. The "*" denotes a p-value <0.05.

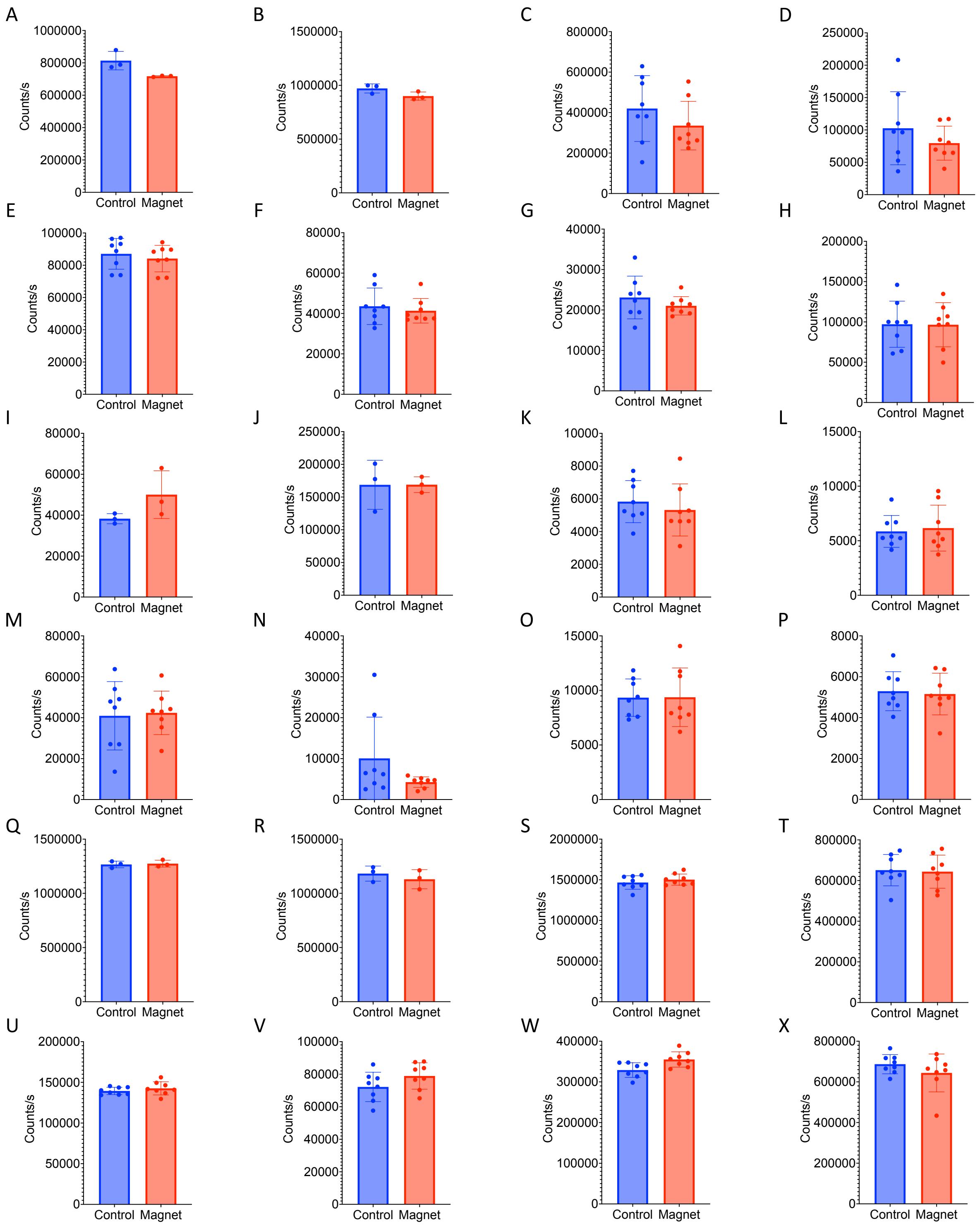

Supplemental Figure 4: Ganciclovir mediated cell death in 4T1 cells.
Cells expressed the EPG split HSV1-TK construct (A-H), HSV1-TK construct (I-P), or were mock transfected (Q-X) and cell viability in either a magnetic stimulated or control conditions.

Statistical analysis of the HSV1-TK experiments:

The experiment includes 8 replicates from which we observed that the average cell growth was inhibited when under magnetic influence compared to the non-magnetic condition, and compared to each of 8 replicates of controls expecting cell growth, and cell death. There are 2 questions about the statistical significance of this observation: 1) "How likely is it to again observe the 8 replicate outcomes of reduced average cell growth if the magnetic condition actually had no effect?" 2) "How likely is it to again observe in each replicate the particular difference of averages, if the magnetic condition actually had no effect?" These can be thought of as condition and replicate significance testing, respectively.

For the condition significance testing, we investigated how likely it is to observe all 8 replicates of average cell growth showing inhibition if the experimental magnetic condition had no effect. To test this, we simulated how often we observe all 8 replicates with inhibited cell growth if we were to perform the growth and death control replicates many more times. This test is simulated because more replicate data becomes costly and laborious to gather. To simulate more control replicates we sampled our existing control replicates with replacement, counting how many samples of 8 contain all 8 showing inhibited cell growth. By chance, we observed our experimental results from the simulated sampling of the control conditions with a probability of 0.00039 (see Equation 1). This is sufficiently low to suggest significance of inhibition between replicate conditions. Probability density for the binomial distribution is shown in Equation 1, where n is the number of trials, p is the probability of success, and N is the number of successes.

$$P(N) = \binom{n}{N} p^N (1-p)^{n-N}$$

Equation: 1. Our control conditions both have 3 replicates with average inhibited cell growth or death, and 5 replicates with invigorated cell growth or death. Assuming this outcome was the most common outcome to observe of the underlying distribution, then the probability of inhibition in the controls then becomes 3/8 or p=0.375, and N=8. Using the binomial formula yields P(8)=0.00039.

For the replicate significance testing, we investigated how likely it is to observe, for each replicate, the particular difference of means if the experimental magnetic condition had no effect and the data for both conditions had come from the same distribution. To test this, we used a Randomization Test[2] wherein the data comprising each replicate for both magnetic and non-magnetic conditions are assumed to originate from the same source. We randomize the "magnetic" and "non-magnetic" labels from the collected data for each replicate, then recalculate the difference of means. When performed many times (1,000,000), this process creates a distribution of differences, from which we can calculate how often the actual observed means difference or better can arise. All experimental condition replicates (EPG-HSV1-TK) showed statistical significance that the observed difference of means was far greater than

the 95% confidence interval of the mean in which the differences of the means were sampled from randomized data assuming no effect of the magnetic condition (Figures S5-S7).

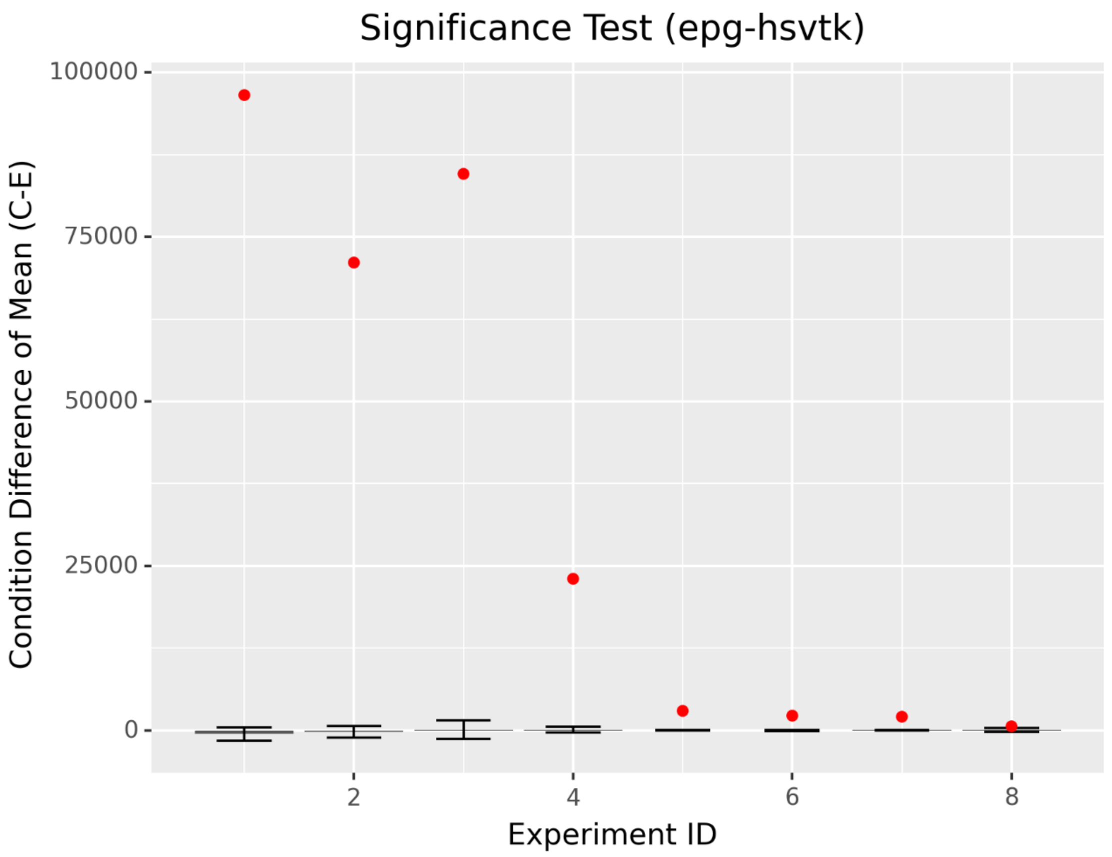

Supplemental Figure 5: Significance Testing of EPG-HSV1-TK construct for Ganciclovir mediated cell death in 4T1 cells. Results of 1,000,000 iterations of randomizing the labels to generate differences in the mean. Each experimental replicate was tested and showed each mean is greater than the 95% confidence interval.

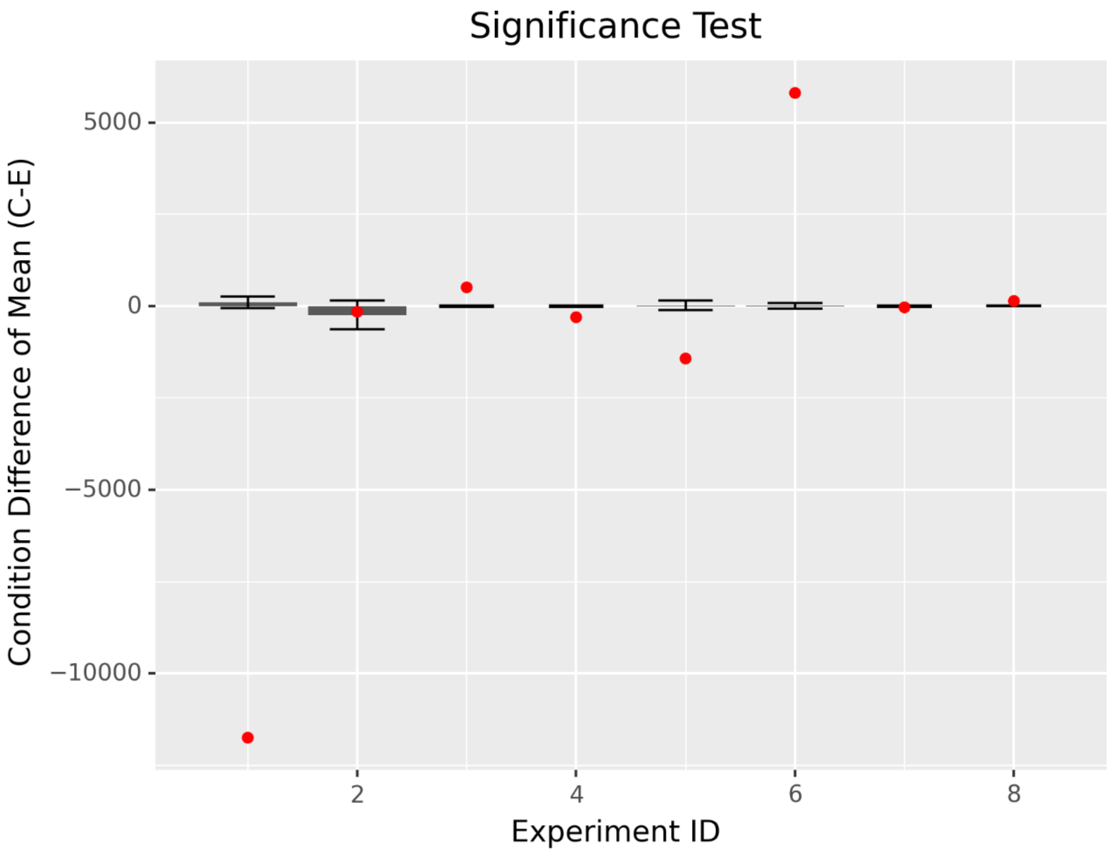

Supplemental Figure 6: Significance Testing of HSV1-TK construct for Ganciclovir mediated cell death in 4T1 cells. Results of 1,000,000 iterations of randomizing the labels to generate differences in the mean. Each experimental replicate was tested and did not show consistent difference in means.

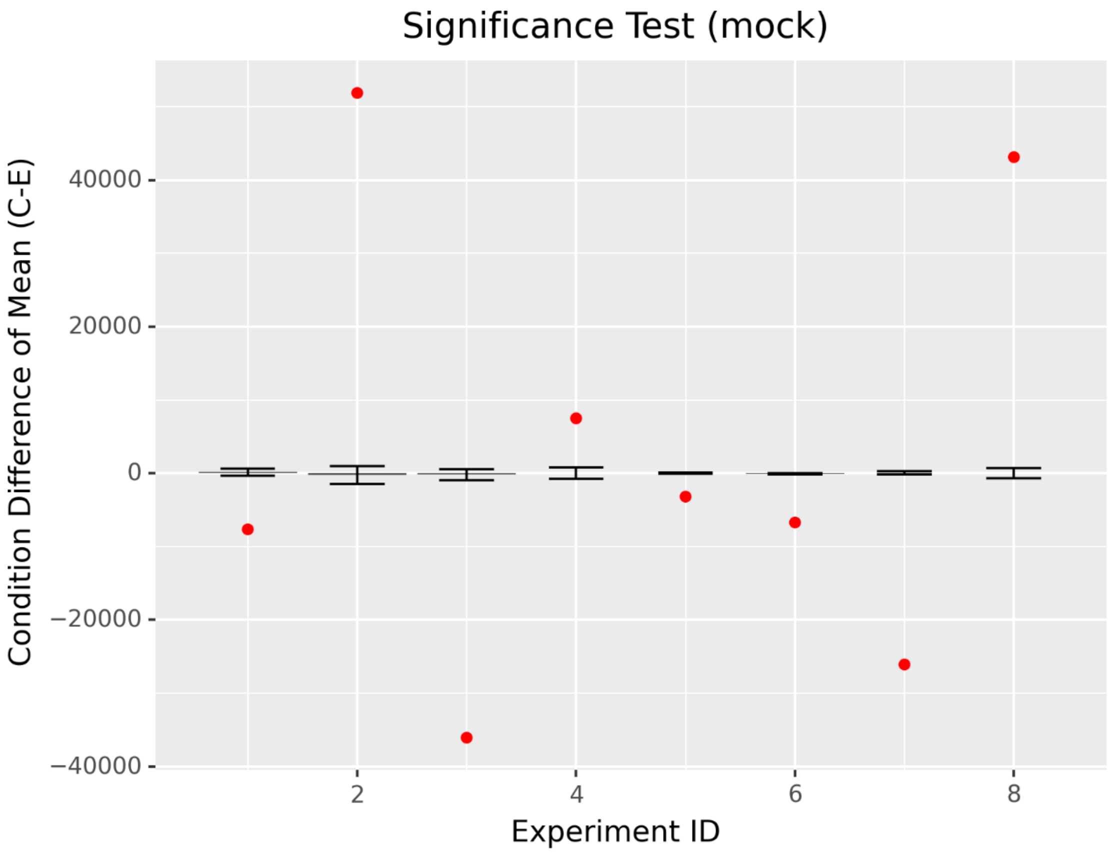

Supplemental Figure 7: Significance Testing of Mock transfected cells for Ganciclovir mediated cell death in 4T1 cells. Results of 1,000,000 iterations of randomizing the labels to generate differences in the mean. Each experimental replicate was tested and did not show consistent difference in means.

Constructs

EPG BRET Constructs
EPG BRET- Vector: pcDNA 3.1 Promoter: CMV
Start-NanoLuc-EPG-mVenus-Stop
ATGGTCTTCACACTCGAAGATTTCGTTGGGGACTGGCGACAGACAGCCGGCTACAACCTGGACCAAGTCC
TTGAACAGGGAGGTGTGTCCAGTTTGTTTCAGAATCTCGGGGTGTCCGTAACTCCGATCCAAAGGATTGT
CCTGAGCGGTGAAAATGGGCTGAAGATCGACATCCATGTCATCATCCCGTATGAAGGTCTGAGCGGCGAC
CAAATGGGCCAGATCGAAAAAATTTTTAAGGTGGTGTACCCTGTGGATGATCATCACTTTAAGGTGATCC
TGCACTATGGCACACTGGTAATCGACGGGGTTACGCCGAACATGATCGACTATTTCGGACGGCCGTATGA
AGGCATCGCCGTGTTCGACGGCAAAAAGATCACTGTAACAGGGACCCTGTGGAACGGCAACAAAATTATC
GACGAGCGCCTGATCAACCCCGACGGCTCCCTGCTGTTCCGAGTAACCATCAACGGAGTGACCGGCTGGC
GGCTGTGCGAACGCATTCTGGCGATGAAGTGTGTACTTTTGGGATTCGCAGCAGTGATCGGATTCTTCGC
GATCGCGGAGTCTCTTACCTGTAACACATGCTCAGTGAGTCTGATTGGAATATGTCTGAATCCCGCAACA
GCGACTTGCTCCACCAACACATCCGTCTGCACCACAGGAAGAGCCAGTTTCACGGGCGTCCTCGGCTTCC
TGGGCTTCAACTCCCAGGGCTGCACGGAGGGAGCTCAGTGTAATGGCACCGTGTCCGGGTCCATCCTGGG
TGCGTCGTACACGGTCACTCAAACCTGCTGCAGCACAAACAACTGCAACCCCGTGACCAGCGGCGCCTCC
TACGTCCAGATCTCCGTCAGCGCGGCCCTGAGCGCCGCCCTGCTGGCCTGCGTCTGGGGCCAGTCCGTCT
ACATGGTGAGCAAGGGCGAGGAGCTGTTCACCGGGGTGGTGCCCATCCTGGTCGAGCTGGACGGCGACGT
AAACGGCCACAAGTTCAGCGTGTCCGGCGAGGGCGAGGGCGATGCCACCTACGGCAAGCTGACCCTGAAG
CTGATCTGCACCACCGGCAAGCTGCCCGTGCCCTGGCCCACCCTCGTGACCACCCTGGGCTACGGCCTGC
AGTGCTTCGCCCGCTACCCCGACCACATGAAGCAGCACGACTTCTTCAAGTCCGCCATGCCCGAAGGCTA
CGTCCAGGAGCGCACCATCTTCTTCAAGGACGACGGCAACTACAAGACCCGCGCCGAGGTGAAGTTCGAG
GGCGACACCCTGGTGAACCGCATCGAGCTGAAGGGCATCGACTTCAAGGAGGACGGCAACATCCTGGGGC
ACAAGCTGGAGTACAACTACAACAGCCACAACGTCTATATCACCGCCGACAAGCAGAAGAACGGCATCAA
GGCCAACTTCAAGATCCGCCACAACATCGAGGACGGCGGCGTGCAGCTCGCCGACCACTACCAGCAGAAC
ACCCCCATCGGCGACGGCCCCGTGCTGCTGCCCGACAACCACTACCTGAGCTACCAGTCCGCCCTGAGCA
AAGACCCCAACGAGAAGCGCGATCACATGGTCCTGCTGGAGTTCGTGACCGCCGCCGGGATCACTCTCGG
CATGGACGAGCTGTACAAGTAA

Tandem EPG BRET- Vector: pcDNA 3.1 Promoter: CMV
Start-NanoLuc-EPG-EPG-mVenus-Stop
ATGGTCTTCACACTCGAAGATTTCGTTGGGGACTGGCGACAGACAGCCGGCTACAACCTGGACCAAGTCC
TTGAACAGGGAGGTGTGTCCAGTTTGTTTCAGAATCTCGGGGTGTCCGTAACTCCGATCCAAAGGATTGT
CCTGAGCGGTGAAAATGGGCTGAAGATCGACATCCATGTCATCATCCCGTATGAAGGTCTGAGCGGCGAC
CAAATGGGCCAGATCGAAAAAATTTTTAAGGTGGTGTACCCTGTGGATGATCATCACTTTAAGGTGATCC
TGCACTATGGCACACTGGTAATCGACGGGGTTACGCCGAACATGATCGACTATTTCGGACGGCCGTATGA
AGGCATCGCCGTGTTCGACGGCAAAAAGATCACTGTAACAGGGACCCTGTGGAACGGCAACAAAATTATC
GACGAGCGCCTGATCAACCCCGACGGCTCCCTGCTGTTCCGAGTAACCATCAACGGAGTGACCGGCTGGC
GGCTGTGCGAACGCATTCTGGCGATGAAGTGTGTACTTTTGGGATTCGCAGCAGTGATCGGATTCTTCGC
GATCGCGGAGTCTCTTACCTGTAACACATGCTCAGTGAGTCTGATTGGAATATGTCTGAATCCCGCAACA
GCGACTTGCTCCACCAACACATCCGTCTGCACCACAGGAAGAGCCAGTTTCACGGGCGTCCTCGGCTTCC
TGGGCTTCAACTCCCAGGGCTGCACGGAGGGAGCTCAGTGTAATGGCACCGTGTCCGGGTCCATCCTGGG
TGCGTCGTACACGGTCACTCAAACCTGCTGCAGCACAAACAACTGCAACCCCGTGACCAGCGGCGCCTCC
TACGTCCAGATCTCCGTCAGCGCGGCCCTGAGCGCCGCCCTGCTGGCCTGCGTCTGGGGCCAGTCCGTCT

ACATGAAGTGTGTACTTTTGGGATTCGCAGCAGTGATCGGATTCTTCGCGATCGCGGAGTCTCTTACCTG
TAACACATGCTCAGTGAGTCTGATTGGAATATGTCTGAATCCCGCAACAGCGACTTGCTCCACCAACACA
TCCGTCTGCACCACAGGAAGAGCCAGTTTCACGGGCGTCCTCGGCTTCCTGGGCTTCAACTCCCAGGGCT
GCACGGAGGGAGCTCAGTGTAATGGCACCGTGTCCGGGTCCATCCTGGGTGCGTCGTACACGGTCACTCA
AACCTGCTGCAGCACAAACAACTGCAACCCCGTGACCAGCGGCGCCTCCTACGTCCAGATCTCCGTCAGC
GCGGCCCTGAGCGCCGCCCTGCTGGCCTGCGTCTGGGGCCAGTCCGTCTACATGGTGAGCAAGGGCGAGG
AGCTGTTCACCGGGGTGGTGCCCATCCTGGTCGAGCTGGACGGCGACGTAAACGGCCACAAGTTCAGCGT
GTCCGGCGAGGGCGAGGGCGATGCCACCTACGGCAAGCTGACCCTGAAGCTGATCTGCACCACCGGCAAG
CTGCCCGTGCCCTGGCCCACCCTCGTGACCACCCTGGGCTACGGCCTGCAGTGCTTCGCCCGCTACCCCG
ACCACATGAAGCAGCACGACTTCTTCAAGTCCGCCATGCCCGAAGGCTACGTCCAGGAGCGCACCATCTT
CTTCAAGGACGACGGCAACTACAAGACCCGCGCCGAGGTGAAGTTCGAGGGCGACACCCTGGTGAACCGC
ATCGAGCTGAAGGGCATCGACTTCAAGGAGGACGGCAACATCCTGGGGCACAAGCTGGAGTACAACTACA
ACAGCCACAACGTCTATATCACCGCCGACAAGCAGAAGAACGGCATCAAGGCCAACTTCAAGATCCGCCA
CAACATCGAGGACGGCGGCGTGCAGCTCGCCGACCACTACCAGCAGAACACCCCCATCGGCGACGGCCCC
GTGCTGCTGCCCGACAACCACTACCTGAGCTACCAGTCCGCCCTGAGCAAAGACCCCAACGAGAAGCGCG
ATCACATGGTCCTGCTGGAGTTCGTGACCGCCGCCGGGATCACTCTCGGCATGGACGAGCTGTACAAGTA
A

EPG IRES BRET- Vector: pcDNA 3.1 Promoter: CMV

Start- EPG-NanoLuc-IRES-EPG-mVenus-Stop

ATGAAGTGTGTACTTTTGGGATTCGCAGCAGTGATCGGATTCTTCGCGATCGCGGAGTCTCTTACCTGTA
ACACATGCTCAGTGAGTCTGATTGGAATATGTCTGAATCCCGCAACAGCGACTTGCTCCACCAACACATC
CGTCTGCACCACAGGAAGAGCCAGTTTCACGGGCGTCCTCGGCTTCCTGGGCTTCAACTCCCAGGGCTGC
ACGGAGGGAGCTCAGTGTAATGGCACCGTGTCCGGGTCCATCCTGGGTGCGTCGTACACGGTCACTCAAA
CCTGCTGCAGCACAAACAACTGCAACCCCGTGACCAGCGGCGCCTCCTACGTCCAGATCTCCGTCAGCGC
GGCCCTGAGCGCCGCCCTGCTGGCCTGCGTCTGGGGCCAGTCCGTCTACATGGTCTTCACACTCGAAGAT
TTCGTTGGGGACTGGCGACAGACAGCCGGCTACAACCTGGACCAAGTCCTTGAACAGGGAGGTGTGTCCA
GTTTGTTTCAGAATCTCGGGGTGTCCGTAACTCCGATCCAAAGGATTGTCCTGAGCGGTGAAAATGGGCT
GAAGATCGACATCCATGTCATCATCCCGTATGAAGGTCTGAGCGGCGACCAAATGGGCCAGATCGAAAAA
ATTTTTAAGGTGGTGTACCCTGTGGATGATCATCACTTTAAGGTGATCCTGCACTATGGCACACTGGTAA
TCGACGGGGTTACGCCGAACATGATCGACTATTTCGGACGGCCGTATGAAGGCATCGCCGTGTTCGACGG
CAAAAAGATCACTGTAACAGGGACCCTGTGGAACGGCAACAAAATTATCGACGAGCGCCTGATCAACCCC
GACGGCTCCCTGCTGTTCCGAGTAACCATCAACGGAGTGACCGGCTGGCGGCTGTGCGAACGCATTCTGG
CCCCCTCTCCCTCCCCCCCCCCTAACGTTACTGGCCGAAGCCGCTTGGAATAAGGCCGGTGTGCGTTTGT
CTATATGTTATTTTCCACCATATTGCCGTCTTTTGGCAATGTGAGGGCCCGGAAACCTGGCCCTGTCTTC
TTGACGAGCATTCCTAGGGGTCTTTCCCCTCTCGCCAAAGGAATGCAAGGTCTGTTGAATGTCGTGAAGG
AAGCAGTTCCTCTGGAAGCTTCTTGAAGACAAACAACGTCTGTAGCGACCCTTTGCAGGCAGCGGAACCC
CCCACCTGGCGACAGGTGCCTCTGCGGCCAAAAGCCACGTGTATAAGATACACCTGCAAAGGCGGCACAA
CCCCAGTGCCACGTTGTGAGTTGGATAGTTGTGGAAAGAGTCAAATGGCTCTCCTCAAGCGTATTCAACA
AGGGGCTGAAGGATGCCCAGAAGGTACCCATTGTATGGGATCTGATCTGGGGCCTCGGTGCACATGCTT
TACATGTGTTTAGTCGAGGTTAAAAAACGTCTAGGCCCCCCGAACCACGGGGACGTGGTTTTCCTTTGAA
AAACACGATGATAATGGCCACAACCATGAAGTGTGTACTTTTGGGATTCGCAGCAGTGATCGGATTCTTC
GCGATCGCGGAGTCTCTTACCTGTAACACATGCTCAGTGAGTCTGATTGGAATATGTCTGAATCCCGCAA
CAGCGACTTGCTCCACCAACACATCCGTCTGCACCACAGGAAGAGCCAGTTTCACGGGCGTCCTCGGCTT
CCTGGGCTTCAACTCCCAGGGCTGCACGGAGGGAGCTCAGTGTAATGGCACCGTGTCCGGGTCCATCCTG

GGTGCGTCGTACACGGTCACTCAAACCTGCTGCAGCACAAACAACTGCAACCCCGTGACCAGCGGCGCCT
CCTACGTCCAGATCTCCGTCAGCGCGGCCCTGAGCGCCGCCCTGCTGGCCTGCGTCTGGGGCCAGTCCGT
CTACATGGTGAGCAAGGGCGAGGAGCTGTTCACCGGGGTGGTGCCCATCCTGGTCGAGCTGGACGGCGAC
GTAAACGGCCACAAGTTCAGCGTGTCCGGCGAGGGCGAGGGCGATGCCACCTACGGCAAGCTGACCCTGA
AGCTGATCTGCACCACCGGCAAGCTGCCCGTGCCCTGGCCCACCCTCGTGACCACCCTGGGCTACGGCCT
GCAGTGCTTCGCCCGCTACCCCGACCACATGAAGCAGCACGACTTCTTCAAGTCCGCCATGCCCGAAGGC
TACGTCCAGGAGCGCACCATCTTCTTCAAGGACGACGGCAACTACAAGACCCGCGCCGAGGTGAAGTTCG
AGGGCGACACCCTGGTGAACCGCATCGAGCTGAAGGGCATCGACTTCAAGGAGGACGGCAACATCCTGGG
GCACAAGCTGGAGTACAACTACAACAGCCACAACGTCTATATCACCGCCGACAAGCAGAAGAACGGCATC
AAGGCCAACTTCAAGATCCGCCACAACATCGAGGACGGCGGCGTGCAGCTCGCCGACCACTACCAGCAGA
ACACCCCCATCGGCGACGGCCCCGTGCTGCTGCCCGACAACCACTACCTGAGCTACCAGTCCGCCCTGAG
CAAAGACCCCAACGAGAAGCGCGATCACATGGTCCTGCTGGAGTTCGTGACCGCCGCCGGGATCACTCTC
GGCATGGACGAGCTGTACAAGTAA

EPG split NanoLuc- Vector: pET101 Promoter: T7laq

Start-N-Terminal NanoLuc-3AA Linker-EPG-3AA Linker-C-Terminal NanoLuc-Stop

ATGGTCTTCACACTCGAAGATTTCGTTGGGGACTGGCGACAGACAGCCGGCTACAACCTGGACCAAGTCC
TTGAACAGGGAGGTGTGTCCAGTTTGTTTCAGAATCTCGGGGTGTCCGTAACTCCGATCCAAAGGATTGT
CCTGAGCGGTGAAAATGGGCTGAAGATCGACATCCATGTCATCATCCCGTATGAGGAGGTAGTAAGTGTG
TACTTTTGGGATTCGCAGCAGTGATCGGATTCTTCGCGATCGCGGAGTCTCTTACCTGTAACACATGCTC
AGTGAGTCTGATTGGAATATGTCTGAATCCCGCAACAGCGACTTGCTCCACCAACACATCCGTCTGCACC
ACAGGAAGAGCCAGTTTCACGGGCGTCCTCGGCTTCCTGGGCTTCAACTCCCAGGGCTGCACGGAGGGAG
CTCAGTGTAATGGCACCGTGTCCGGGTCCATCCTGGGTGCGTCGTACACGGTCACTCAAACCTGCTGCAG
CACAAACAACTGCAACCCCGTGACCAGCGGCGCCTCCTACGTCCAGATCTCCGTCAGCGCGGCCCTGAGC
GCCGCCCTGCTGGCCTGCGTCTGGGGCCAGTCCGTCTACGGAGGTAGTGGTCTGAGCGGCGACCAAATGG
GCCAGATCGAAAAAATTTTTAAGGTGGTGTACCCTGTGGATGATCATCACTTTAAGGTGATCCTGCACTA
TGGCACACTGGTAATCGACGGGGTTACGCCGAACATGATCGACTATTTCGGACGGCCGTATGAAGGCATC
GCCGTGTTCGACGGCAAAAAGATCACTGTAACAGGGACCCTGTGGAACGGCAACAAAATTATCGACGAGC
GCCTGATCAACCCCGACGGCTCCCTGCTGTTCCGAGTAACCATCAACGGAGTGACCGGCTGGCGGCTGTG
CGAACGCATTCTGGCGTAA

EPG-HaloTag- Vector: pcDNA 3.1 Promoter: CMV

EPG Signal sequence-HaloTag-Linker-TEV site-Linker-EPG-3xFLAG

ATGAAGTGTGTACTTTTGGGATTCGCAGCAGTGATCGGATTCTTCGCGATCGCGGAGTCTATGGCAGAAA
TCGGTACTGGCTTTCCATTCGACCCCCATTATGTGGAAGTCCTGGGCGAGCGCATGCACTACGTCGATGT
TGGTCCGCGCGATGGCACCCCTGTGCTGTTCCTGCACGGTAACCCGACCTCCTCCTACGTGTGGCGCAAC
ATCATCCCGCATGTTGCACCGACCCATCGCTGCATTGCTCCAGACCTGATCGGTATGGGCAAATCCGACA
AACCAGACCTGGGTTATTTCTTCGACGACCACGTCCGCTTCATGGATGCCTTCATCGAAGCCCTGGGTCT
GGAAGAGGTCGTCCTGGTCATTCACGACTGGGGCTCCGCTCTGGGTTTCCACTGGGCCAAGCGCAATCCA
GAGCGCGTCAAAGGTATTGCATTTATGGAGTTCATCCGCCCTATCCCGACCTGGGACGAATGGCCAGAAT
TTGCCCGCGAGACCTTCCAGGCCTTCCGCACCACCGACGTCGGCCGCAAGCTGATCATCGATCAGAACGT
TTTTATCGAGGGTACGCTGCCGATGGGTGTCGTCCGCCCGCTGACTGAAGTCGAGATGGACCATTACCGC
GAGCCGTTCCTGAATCCTGTTGACCGCGAGCCACTGTGGCGCTTCCCAAACGAGCTGCCAATCGCCGGTG
AGCCAGCGAACATCGTCGCGCTGGTCGAAGAATACATGGACTGGCTGCACCAGTCCCCTGTCCCGAAGCT
GCTGTTCTGGGGCACCCCAGGCGTTCTGATCCCACCGGCCGAAGCCGCTCGCCTGGCCAAAAGCCTGCCT

AACTGCAAGGCTGTGGACATCGGCCCGGGTCTGAATCTGCTGCAAGAAGACAACCCGGACCTGATCGGCA
GCGAGATCGCGCGCTGGCTGTCGACGCTCGAGATTTCCGGCGAGCCAACCACTGAGGATCTGTACTTTCA
GAGCGATAACCTTACCTGTAACACATGCTCAGTGAGTCTGATTGGAATATGTCTGAATCCCGCAACAGCG
ACTTGCTCCACCAACACATCCGTCTGCACCACAGGAAGAGCCAGTTTCACGGGCGTCCTCGGCTTCCTGG
GCTTCAACTCCCAGGGCTGCACGGAGGGAGCTCAGTGTAATGGCACCGTGTCCGGGTCCATCCTGGGTGC
GTCGTACACGGTCACTCAAACCTGCTGCAGCACAAACAACTGCAACCCCGTGACCAGCGGCGCCTCCTAC
GTCCAGATCTCCGTCAGCGCGGCCCTGAGCGCCGCCCTGCTGGCCTGCGTCTGGGGCCAGTCCGTCTACG
ACTACAAGGATGACGATGACAAGGATTACAAAGACGACGATGATAAGGACTATAAGGATGATGACGACAA
A

EPG Split APEX2 Vector: pcDNA 3.1 Promoter: CMV

Start-AP Fragment-Linker-EPG-Linker-EX Fragment-Stop

ATGGGGAAATCATACCCAACAGTGTCCGCAGACTACCAGGATGCCGTGGAGAAGCCAAGAAGAGGCTGG
GAGGGTTTATCGCAGAAAAGAGGTGCGCACCTCTGATGCTGAGACTGGCTTTCCACAGCGCAGGCACCTT
TGACAAGAGAACCAAAACAGGCGGACCCTTTGGAACAATCAGGTACCCCGCTGAACTGGCACATAGTGCC
AACAGCGGGCTGGACATCGCCGTGCGGCTGCTGGAACCTCTGAAAGCAGAGTTCCCAATTCTGTCCTACG
CCGATTTTTATCAGCTGGCAGGAGTGGTCGCTGTGGAGGTCACTGGGGGCCCCAAGGTGCCTTTTCACCC
AGGACGGGAGGACAAGCCAGAACTACCTCCAGAGGGGCGCCTGCCAGATCCGACAAAGGGCTCCGACCAT
CTGCGAGATGTGTTTGGGAAAGCTATGGGCCTGACTGACCAGGATATCGTCGCACTGTCTGGAGGGCACA
CCCTTGGCGCCGCTCATAAGGAAAGGTCAGGCTTCGAGGGACCCTGGACAAGCAACCCCCTGGTTTTCGA
CAATTCTTACTTTACTGAACTGCTGAGTGGAGAGAAGGAAAAGGGCTCGGGCTCGACCTCGAAGTGTGTA
CTTTTGGGATTCGCAGCAGTGATCGGATTCTTCGCGATCGCGGAGTCTCTTACCTGTAACACATGCTCAG
TGAGTCTGATTGGAATATGTCTGAATCCCGCAACAGCGACTTGCTCCACCAACACATCCGTCTGCACCAC
AGGAAGAGCCAGTTTCACGGGCGTCCTCGGCTTCCTGGGCTTCAACTCCCAGGGCTGCACGGAGGGAGCT
CAGTGTAATGGCACCGTGTCCGGGTCCATCCTGGGTGCGTCGTACACGGTCACTCAAACCTGCTGCAGCA
CAAACAACTGCAACCCCGTGACCAGCGGCGCCTCCTACGTCCAGATCTCCGTCAGCGCGGCCCTGAGCGC
CGCCCTGCTGGCCTGCGTCTGGGGCCAGTCCGTCTACGGATCCAAGGGCTCGGGCTCGACCTCGGGCTCG
GGCGGGCTGCTGCAGCTGCCCAGCGACAAAGCCCTGCTGTCCGATCCCGTGTTCAGACCTCTGGTCGATA
AGTATGCAGCCGACGAGGATGCTTTTTTCGCAGATTACGCAGAAGCACATCAGAAGCTGTCAGAACTGGG
ATTTGCCGACGCCTAA

EPG Split HSVTK Vector: pTwist CMV Puro Promoter: CMV

Start-N-Terminal HSV-TK-Linker-EPG-Linker-C-Terminal HSV-TK-Stop

ATGGCCTTGACGCCCCAGGGTGCCGAGCCCCAGAGCAACGCGGGCCCACGACCCCATATCGGGGAAACGT
TATTTACCCTGTTTCGGGCCCCCGAGTTGCTGGCCCCCAACGGCGACCTGTACAACGTGTTTGCCTGGGC
CTTGGACGTCTTGGCCAAACGCCTCCGTCCCATGCACGTCTTTATCCTGGATTACGACCAATCGCCCGCC
GGCTGCCGGGACGCCCTGCTGCAACTTACCTCCGGGATGGTCCAGACCCATGTCACCACCCCAGGCTCCA
TACCGACGATCTGCGACCTGGCGCGCACGTTTGCCCGGGAGATGGGGGAGGCTCACGGAGGTGGTGGAAG
TAAGTGTGTACTTTTGGGATTCGCAGCAGTGATCGGATTCTTCGCGATCGCGGAGTCTCTTACCTGTAAC
ACATGCTCAGTGAGTCTGATTGGAATATGTCTGAATCCCGCAACAGCGACTTGCTCCACCAACACATCCG
TCTGCACCACAGGAAGAGCCAGTTTCACGGGCGTCCTCGGCTTCCTGGGCTTCAACTCCCAGGGCTGCAC
GGAGGGAGCTCAGTGTAATGGCACCGTGTCCGGGTCCATCCTGGGTGCGTCGTACACGGTCACTCAAACC
TGCTGCAGCACAAACAACTGCAACCCCGTGACCAGCGGCGCCTCCTACGTCCAGATCTCCGTCAGCGCGG
CCCTGAGCGCCGCCCTGCTGGCCTGCGTCTGGGGCCAGTCCGTCTACCCCGCTCCAGCACCTGCTTCGTA
CCCCTGCCATCAACACGCGTCTGCGTTCGACCAGGCTGCGCGTTCTCGCGGCCATAGCAACCGACGTACG

GCGTTGCGCCCTCGCCGGCAGCAAGAAGCCACGGAAGTCCGCCCGGAGCAGAAAATGCCCACGCTACTGC
GGGTTTATATAGACGGTCCCCACGGGATGGGGAAAACCACCACCACGCAACTGCTGGTGGCCCTGGGTTC
GCGCGACGATATCGTCTACGTACCCGAGCCGATGACTTACTGGCGGGTGCTGGGGGCTTCCGAGACAATC
GCGAACATCTACACCACACAACACCGCCTCGACCAGGGTGAGATATCGGCCGGGGACGCGGCGGTGGTAA
TGACAAGCGCCCAGATAACAATGGGCATGCCTTATGCCGTGACCGACGCCGTTCTGGCTCCTCATATCGG
GGGGGAGGCTGGGAGCTCACATGCCCCGCCCCCGGCCCTCACCATCTTCCTCGACCGCCATCCCATCGCC
TTCATGCTGTGCTACCCGGCCGCGCGGTACCTTATGGGCAGCATGACCCCCCAGGCCGTGCTGGCGTTCG
TGGCCCTCATCCCGCCGACCTTGCCCGGCACAAACATCGTGTTGGGGGCCCTTCCGGAGGACAGACACAT
CGACCGCCTGGCCAAACGCCAGCGCCCCGGTGAGCGGCTTGACCTGGCTATGCTGGCCGCGATTCGCCGC
GTTTACGGGCTACTTGCCAATACGGTGCGGTATCTGCAGTGCGGCGGGTCGTGGCGGGAGGATTGGGGAC
AGCTTTCGGGGACGTGA

1. Petersen, E.D. et al. Bioluminescent Genetically Encoded Glutamate Indicators for Molecular Imaging of Neuronal Activity. *ACS Synthetic Biology* (2023).
2. Manly, B.F.J. Randomization Tests, 4th Edition by Eugene S. Edgington, Patrick Onghena.
